# Tuberculin skin tests following Bacille Calmette Guerin vaccination in Africa: a protocol for systematic review and meta-analysis

**DOI:** 10.11604/pamj.2022.41.12.30535

**Published:** 2022-01-05

**Authors:** Chinonyelum Thecla Ezeonu, Richard Chinaza Ikeagwulonu, Uzoma Vivian Asiegbu, Dorathy Chinwe Obu, Datonye Christopher Briggs

**Affiliations:** 1 Department of Pediatrics, Alex Ekwueme Federal University Teaching Hospital Abakaliki, Ebonyi State, Nigeria,; 2 Department of Medical Laboratory Science, Alex Ekwueme Federal University Teaching Hospital Abakaliki, Ebonyi State, Nigeria,; 3 Department of Pediatrics, Rivers State University Teaching Hospital, Port Harcourt, Rivers State, Nigeria

**Keywords:** Tuberculosis, *Mycobacterium tuberculosis*, Bacille Calmette Guerin, tuberculin skin test, tuberculin unit, purified protein derivatives

## Abstract

Tuberculin reactivity following tuberculin skin test which is the most common measure of the effect of the Bacille Calmette Guerin (BCG) vaccination has remained complex. This protocol is aimed to assess the effect of BCG vaccine on tuberculin skin test in Africa as a guide to better understanding or resolving this complexity. A search strategy is developed using MeSH, key words, text words, and entry terms. Five databases will be searched, including PubMed, African Journals Online (AJOL), Google Scholar, Research Gate, and Cochrane Library. Only observational studies conducted in Sub-Saharan Africa and retrievable in English language will be included. The primary measurable outcome of this study is pooled prevalence of positive tuberculin skin tests. Secondary outcomes are factors that influence Tuberculin Skin Test (TST) positivity such as BCG type, the dose of BCG, age at BCG vaccinates, time/interval between BCG vaccination and TST, and type of tuberculin unit used for TST. Identified studies will be screened and selected based on inclusion criteria. Data will be extracted into Zotero, Microsoft Excel and CMA software. Both quality scores and the risk of bias for individual studies will be reported. Studies will be assessed for methodological, clinical, and statistical heterogeneity. Funnel Plots will be used for assessing publication bias. The results will be presented in tabular format in addition to a narrative synthesis. The pooled prevalence of effect of BCG vaccine on TST in Africa will be examined in relation to factors that influence TST positivity.

## Introduction

Tuberculosis is a chronic disease posing a public health challenge particularly in resource poor countries [1]. In 2017, about 10 million new cases of TB with 1.6 million deaths were reported globally [2]. However, one surest way of preventing TB globally had been through vaccination and Bacille Calmette Guerin (BCG) vaccine has been widely used across continents. The BCG vaccine is routinely given to protect individuals, especially children, from serious forms of tuberculosis disease [3]. In Africa and in most TB endemic countries, the vaccine is usually administered around birth to prevent severe TB in infants. Importantly, asymptomatic latent tuberculosis infection can be detected through performance of tuberculin skin test (TST), a procedure which utilizes a purified protein derivative (PPD) of heat killed live cultures of mycobacteria injected intra-dermally into the forearm to demonstrate delayed hypersensitivity response to the PPD, which fully occurs within 48-72 hours [4].

Tuberculin reactivity which can either be positive or negative following tuberculin skin test has been the most common measure of the effect of the BCG vaccination. Positive tuberculin skin test has been reported in previously BCG vaccinated individuals and in circumstances such as recent contact with another person with infectious TB or continual exposure to populations with a high prevalence of TB [5,6]. Conditions such as cross reactivity of the PPD with other non-tuberculous mycobacterial organisms, presence of morbidities such as HIV co-infection, and the immune status of the individuals have been reported to affect TST results [4,7,8]. Vaccination with live viruses such as live-measles, may cause false-negative tuberculin reactions [9] while anergy can occur in individuals with impaired cellular immune function such as those with severe malnutrition or immune-deficiency diseases. Also, the technique of BCG administration, dose, the age at which vaccine was administered, gender, socioeconomic status, degree of TB burden in the community, the people´s genetic background, ethnicity, the manufacturer of the vaccine, the interval between vaccination and testing, have been reported to affect the risk and degree of a reactive PPD tuberculin skin test following BCG vaccination [10-16]. Notwithstanding these confounding effects of BCG on PPD tuberculin test readings, exposure to TB through contacts is more likely to increase positive PPD [17,18], hence, individuals from countries with a high or moderate incidence of TB are more likely to have reactive PPDs than those from countries of low incidence. Nonetheless, reports from various studies have shown conflicting evidence in interpreting a true-positive Tuberculin Skin Test (TST) secondary to an infection and a false-positive test due to a previous vaccination [19,20]. Tuberculin test reading of 10mm or more in the absence of high risk of tuberculosis, has been generally accepted as positive result, in most studies [21,22]. The US Preventive Services Task Force (USPSTF) without any specific recommendation regarding BCG effects on PPD readings, generally states that reactions >10 mm should not be attributed to prior BCG vaccine [23]. Tuberculin skin test measurement of 5mm had been reported among the immune-compromised individuals [4].

In some studies, increased tuberculin reactivity was seen in individuals that received high-dose of the vaccine [24], those whose vaccination or revaccination were given later in life [25-27], and in cases of booster phenomenon [28]. The multipuncture technique has been reported to have significantly resulted in fewer conversions to positive tuberculin skin tests than the intradermal method [29]. In all, the effect of BCG vaccine on tuberculin test reactivity is quite complex making the use of this test to diagnose latent tuberculosis difficult. The uncertainty of the effect of BCG vaccination on TST results and interpretation may affect TB control and treatment. Due to the public health concern of Tuberculosis, it has thus become necessary to conduct a meta-analysis of studies that reported the relationship between TST reactivity in BCG vaccinated and non-vaccinated individuals for better understanding and guide.

**Aim:** the main objective of this review is to assess the effect of Bacille Calmette Guerin vaccine on tuberculin skin test in Africa.

**Study objectives:** 1) to determine the pooled prevalence of positive tuberculin skin tests in Africa. 2) To determine the prevalence of positive tuberculin skin tests in subgroups such as children, adults, previously BCG vaccinated, and non-vaccinated individuals in Africa. 3) To determine the factors that influence TST positivity such as BCG type, the dose of BCG, age at BCG vaccination, time/interval between BCG vaccination and TST, type of tuberculin unit used for TST, the strength of tuberculin unit used for TST, presence, and absence of BCG scar.

**Review questions:** 1) what is the pooled prevalence of positive tuberculin skin tests in Africa? 2) What is the prevalence of positive tuberculin skin tests in subgroups such as children, adults, previously BCG vaccinated, and non-vaccinated individuals? 3) What are the factors that influence TST positivity?

## Methods

**Study design:** this is a protocol for systematic review and meta-analysis of observational studies that reported on the effect of Bacille Calmette Guerin vaccine on tuberculin skin test in Africa. Other types of study designs are excluded including interventional studies, comments, and editorials. There is no time restriction on eligible primary studies.

**Inclusion criteria:** a) observational studies (cohort, cross-sectional, case-control, historical cohort). b) Studies of all years that are published or retrievable in the English language. c) Studies that are available in electronic databases. d) Studies that report the proportion of positive Tuberculin skin test cases (this is the primary outcome measure) and/ or secondary outcome which are factors that influence TST positivity.

**Exclusion criteria:** a) studies without proportion of positive Tuberculin skin test cases. b) Studies that included known contacts of active TB cases. c) Studies that included people with TB infection or disease. d) Narrative reviews, interventional and experimental studies, and letters to the editor e) Studies that are not reported or retrievable in the English language.

**Study characteristics:** the PICOS is as follows: participants are individuals who received tuberculin skin test with or without BCG vaccination in Africa. Intervention: there is no intervention. Comparator: there is no comparator. Outcome: the primary outcome is the proportion of positive tuberculin skin tests. The measurable secondary outcomes are factors that influence TST positivity. Filters used for subgroups are children, adults, and previously BCG vaccinated individuals.

**Information sources:** the search will employ sensitive topic-based strategies designed for each database. The five databases to be searched are PubMed, African Journal Online (AJOL), Google Scholar, Research Gate, and Cochrane Library. The study will retrieve only observational studies written in the English language. There is no time frame or restriction in the inclusion of publications.

**Search strategy:** the search strategy will include MeSH terms, text words, entry words, and keywords. The search strategies that will be used are shown in Table 1.

**Table 1:** search strategy

Database	Search strategy
Pubmed	((((Tuberculin Tests) OR “Tuberculin Test”[Mesh] OR Purified Protein Derivative of Tuberculin) OR “Tuberculin”[Mesh]) AND “BCG Vaccine”[Mesh] OR Calmette Guerin Bacillus Vaccine) AND “Africa”[Mesh]
AJOL	(Tuberculin Tests) OR “Tuberculin Test” OR Purified Protein Derivative of Tuberculin) OR “Tuberculin” AND “BCG Vaccine” OR Calmette Guerin Bacillus Vaccine) AND “Africa”
Google scholar	((((Tuberculin Tests) OR “Tuberculin Test”[Mesh] OR Purified Protein Derivative of Tuberculin) OR “Tuberculin”[Mesh]) AND “BCG Vaccine”[Mesh] OR Calmette Guerin Bacillus Vaccine) AND “Africa”[Mesh]
Research gate	Tuberculin Tests OR “Tuberculin Test” OR Purified Protein Derivative of Tuberculin OR “Tuberculin” AND “BCG Vaccine” OR Calmette Guerin Bacillus Vaccine AND “Africa”
Cochrane Library	Tuberculin Tests OR “Tuberculin Test” OR Purified Protein Derivative of Tuberculin OR “Tuberculin” AND “BCG Vaccine” OR Calmette Guerin Bacillus Vaccine AND “Africa”

**Data extraction:** the data will be extracted and managed using the following tools: i). Zotero software, ii) Microsoft Excel, and iii) Comprehensive Meta-analysis software (CMA) version 3.

**Screening:** studies will be searched using the search strategy. Six levels of data screening will be involved. Level 1: screening to select only observational studies while other study designs are excluded. Level 2: screening of all selected observational studies by their titles and abstracts using MeSH terms, keywords, and entry terms. Level 3: screening of the selected studies by full-text reading, using the same strategy. Level 4: snowballing of the literature using included studies. Level 5: screening for primary and secondary outcomes. Level 6: screening for risk of bias using NIH quality assessment for observational studies and Cochrane risk of bias to strengthen the evidence.

**Selection process and review:** five reviewers are involved in this study. A pair of reviewers will independently screen studies from each database, assessing the studies for inclusion and or exclusion. Conflicts will be resolved by a third independent reviewer. All reviews are blinded. The screening and deduplication will be done in Zotero software. Screened studies will be exported into Microsoft Excel, to be arranged for data analysis. Studies will be selected based on eligibility criteria and primary measurable outcome. Authors of studies with missing data will be contacted via email and telephone.

**Data collection:** the following data items will be extracted from each eligible study into Microsoft Excel: i) First author´s surname, year of publication of the study, year of study, country of study, study design ii) Sample size iii) Type and dose of BCG, age at BCG vaccination, the interval between BCG vaccination and tuberculin skin testing, number of subjects that received BCG vaccine, presence or absence of BCG scar, number of positive or negative TST results, type and strength of tuberculin test/antigen used, response rate/wheal size to tuberculin test at specific diameters, and indication for TST. Others are age group studied, age when TST was given, age when BCG was given, gender, socioeconomic status, means of determining BCG vaccination, the technique for applying and reading the test, the immunological status of the subjects, presence of medical conditions in the studied population such as cancer, Diabetes Mellitus, AIDS Nutritional status and BCG coverage rate. Data from Microsoft Excel will be exported to CMA software for meta-analysis.

**Risk of bias:** the risk of bias in included studies will be accessed for the individual studies using the National Institute of Health (NIH) Quality assessment tool for observational cohort and cross-sectional studies. This will be cross-checked with the Cochrane tool of risk of bias assessment for the strength of the body of evidence. Studies with extreme bias will be excluded after assessment in the following areas: 1. Method of testing and reporting at the outcome level. 2. Reporting of study; prevalence with confidence interval or number of positive cases/sample size, as reported at the outcome level. 3. Heterogeneity will be assessed at the study level. 4. Publication bias will be assessed at the study level.

**Assessment of meta-bias:** to test for heterogeneity Cochrane´s Q value, I^2^, T^2^ will be used. I^2^values of less than 40% will be considered low heterogeneity, values > 40 to ≤75 % moderate while values > 75% are high. The effect sizes to be used are prevalence with a confidence interval (CI, 95%).

**Data synthesis:** both narrative and quantitative analysis will be performed. All studies that report the primary outcome with or without secondary outcomes will be included for systematic review, with all measurable outcomes and sample size reported in a tabular format. Studies with primary indices that can be converted to prevalence will be converted in the CMA software version 3. Quantitative data will include pooled prevalence, standard error and 95% CI. Both random and fixed effect models will be assessed, and the appropriate model will be taken based on the forest plots. Subgroup analysis will be done using categorical factors such as age and BCG vaccination status. Meta-regression will be performed to check for the trend in the prevalence of positive tuberculin skin tests over the years.

## Results

The study selection process will be summarized in a flow diagram according to the PRISMA 2015 Statement and PRISMA-P Checklist. A table of the search strategy in various databases showing text words, MeSH, and entry terms will be included. A list of included studies will be summarized in a table. Quantitative data such as effect size (prevalence), 95% CI, P values, and relative weights assigned to studies and heterogeneity tests will be included in the forest plots. A table of quality scores and risk of bias of each eligible study will be included. Forest plots to show sub-group analysis will be included. A cumulative meta-analysis to check for trends will also be included.

## Discussion

This meta-analysis will address important clinical and epidemiological questions about interpreting the TST in patients with previous BCG vaccination which will help health workers to educate patients when offering treatment for latent tuberculosis infection in a setting of previous BCG vaccination, a positive skin test, and no known TB contact. Large or strongly positive skin tests are most probably due to tuberculosis infection rather than BCG.

**Ethics and dissemination:** ethical approval will not be required since this study will be based on published data. The final report of this study will be published in a peer- reviewed scientific journal.
